# Progress and challenges in the computational prediction of gene function using networks: 2012-2013 update

**DOI:** 10.12688/f1000research.2-230.v1

**Published:** 2013-10-31

**Authors:** Paul Pavlidis, Jesse Gillis

**Affiliations:** 1 Centre for High-Throughput Biology and Department of Psychiatry, University of British Columbia, Vancouver, V6T1Z4, Canada; 2 Stanley Institute for Cognitive Genomics, Cold Spring Harbor Laboratory, Woodbury, NY, 11797, USA

## Abstract

In an opinion published in 2012, we reviewed and discussed our studies of how gene network-based guilt-by-association (GBA) is impacted by confounds related to gene multifunctionality. We found such confounds account for a significant part of the GBA signal, and as a result meaningfully evaluating and applying computationally-guided GBA is more challenging than generally appreciated. We proposed that effort currently spent on incrementally improving algorithms would be better spent in identifying the features of data that do yield novel functional insights. We also suggested that part of the problem is the reliance by computational biologists on gold standard annotations such as the Gene Ontology. In the year since, there has been continued heavy activity in GBA-based research, including work that contributes to our understanding of the issues we raised. Here we provide a review of some of the most relevant recent work, or which point to new areas of progress and challenges.

## Building better networks

One of the problems with network-based approaches we documented in our previous papers ^1– 3^ is their tendency to converge on “easy answers”, by which we mean picking genes as candidates for a given disease or function simply because they are involved in many diseases (multifunctional) or are prominent in the network (e.g., hubs) ^1^. A possible solution would be to tailor the network data to particular contexts. Multifunctional genes would then have less of a dominant role, because fewer of their functions would be relevant to the network, and the network might reflect this. Fortuitously, several studies that improve our understanding of the utility of context-specific networks recently appeared, though they do not address the questions of whether they reduce multifunctionality and node degree biases.

Guan *et al.* (2012) constructed 107 tissue-specific networks for the laboratory mouse to be used in disease-gene prioritization ^4^. They used a combination of training data from Gene Ontology (GO) and tissue-specific expression signatures to customize their networks before moving to predicting disease candidate genes. The networks are not built from tissue-specific data, but various data used in combination, with each given a weight computed using "tissue-specific gold standards". The cross-validation performance improvement was significant but modest across most tasks (appearing to be approximately 0.03 on top of areas under receiver operating characteristic curves (AUROCs) ranging from 0.7 to 0.8).

Magger *et al.* (2012) took a different approach, choosing to construct tissue-specific protein interaction networks by down-weighting edges involving genes not expressed in the given tissue ^5^. Their baseline performances are somewhat higher than Guan *et al.* and also show improvement with tissue-specificity (from a mean AUROC of 0.82 to ~0.88). However, the bulk of this performance improvement comes with simply removing genes not expressed in a given tissue. Magger *et al.* provide some evidence that edges involving such genes are the source of prediction errors, as simply down-ranking the genes after analyzing a generic (non-tissue-specific) network was not as effective. While Magger *et al.* primarily restricted themselves to examining disease-gene associations where the causal gene was judged to be tissue-specific, they did examine the full disease-gene data where gains from tissue-specificity were much more modest (approximately AUROC 0.83 to 0.845). In contrast to the specialized task, node removal and attenuation of unexpressed genes performed particularly badly but only at low false positive rates (FPR). At high FPRs, node removal outperformed other methods, suggesting a trade-off between high precision and low precision prediction.

Piro *et al.* (2012) created co-expression networks from groups of genes expressed in specific mouse brain regions and used these together with other information to predict gene-disease relations from OMIM ^6^. They report that integrating tissue-specific data substantially raises their candidate disease prioritization performance. However, the final performance does not appear to be better than reported using quite old methods (~AUROC of 0.8 overall). Because they do not present results using a comparable “generic” network, it is difficult to tell if anything was gained.

Dowell *et al.* (2013) describe the creation and analysis of a mouse embryonic stem cell (mEPSC) specific gene network, relying on extensive manual curation ^7^. This paper caught our attention in part because Dowell *et al.* acknowledge the potential for node degree bias and other issues, and claim “we address many of these potential pitfalls”. However, we were unable to identify the evidence that their methods do so; indeed, the focus on network hubs combined with very high performance of negative control data (assembled from datasets excluding mESCs), suggests multifunctional biases may have had a role. They suggest the use of cell-type-specific data should “reduce the impact of multi-functional genes”, but do not report whether this was indeed the case. This might have been of value in explaining how their network results were more specific, even if performance was not higher than some of the negative controls overall. If context-specific data reduces generic effects, it is of utility even if it yields no improvements in performance as judged by the usual metrics.

These reports can be considered encouraging, but still leave open the question of whether parsing data into more specific subsets is worthwhile, despite the hopes we expressed last year on this count. The noisiness of biological data may be such that breaking data into smaller bins can cost more in terms of robustness than we gain in terms of specificity. We also note that some earlier approaches combine a wide array of expression data, and treat the data sets as features to be weighted in the prioritization method ^8, 9^. Thus information such as “gene A is expressed in tissue X” may have been implicitly used. Choosing “tissue-specific functions” to assess such approaches is another challenge, and it is unknown if multifunctionality effects are reduced. None of this eliminates the possibility that network specificity provides crucial value, but more data are required.

## Using better controls

The GBA studies discussed in the previous section did not take the opportunity to test the effect of multifunctionality or node degree bias, despite this control being easy to perform. The clearest attempt to control for multifunctionality and node degree of which we are aware is reported by Singh-Blom and colleagues in characterizing their prediction tool, CATAPAULT. Singh-Blom *et al.* conducted an analysis of disease and drug-target genes with a variety of networks and algorithms ^10^. They report that a ranking of genes by multifunctionality (which they refer to as “degree”) performs poorly but not negligibly as a predictor, outperforming some of the methods they tested in cross-validation.

Before we comment further, there are some nuances to how Singh-Blom *et al.* (2013) use the multifunctionality ranking, compared to how we did. First, to avoid confusion the multifunctionality ranking (or a node degree ranking) should not be treated as a “method” for prediction, as it is referred to by Singh-Blom *et al.* It should be considered a null. In addition, the multifunctionality ranking is expected to “perform best” when performance is measured using ROC curves; Singh-Blom *et al.* use something more akin to precision-recall, which tends to obscure the influence of multifunctionality (and node degree), while being more heavily influenced by critical edges. Finally, multifunctionality ranking may be a too-stringent control (when using ROC) because it is literally optimized and performs better than many real algorithms on actual data ^1^; rather, the correlation of the “real” prediction results with multifunctionality ranking is often a more helpful measure.

In any case, the fact that the multifunctionality ranking yields even modest performance in the evaluation scheme of Singh-Blom *et al.* hints that this ranking is also correlated with network node degree (as explained in our work ^1^), and furthermore that such effects will have a strong impact on their results. Accordingly, Singh-Blom *et al.* report that highly prioritized genes tend to have high network node degree. They also report that of the top 10 candidates for eight diseases, almost all were shared among two or more of the diseases, confirming our finding that GBA too often yields “generic” predictions, not function-specific ones. These results, using different algorithms, networks and evaluation metrics than us, provide strong independent support of our claims.

While Singh-Blom *et al.* confirmed some of our key findings, we may differ with them in interpretation, as they argue that the results are unproblematic. We agree with them that non-specific predictions could be correct, but this does not absolve concern about how their methods are actually operating. For example, INSR (insulin receptor) was predicted by CATAPAULT as a leukemia candidate gene, in addition for other diseases (including other cancers and diabetes). We are led to suspect this is at least partly explained by the high node degree of INSR, and not specific “guilt by association” of INSR with known leukemia genes. Can one find literature that connects insulin receptors and leukemia? Of course, since both are highly studied (one can find papers linking insulin receptors or cancer to many things) and the metabolism of cancer cells is of interest from a therapeutic standpoint. We also note that TP53 was predicted to be a diabetes-related gene by CATAPAULT. It remains possible that INSR is a *bona fide* leukemia gene; regardless, we strongly believe that a biologist wanting to use the output of CATAPAULT would also want to know about the specificity of the predictions.

We hope that other researchers interested in why their methods work take the step of attempting to control for “generic” results. Otherwise methodological performance is open to profound misinterpretation as to true utility. This is true even in the case where authors are clearly aware of the potential for problems. For example, Zuberi *et al.* (2013) report that the GeneMANIA edge weight normalization “helps to reduce the impact that the pleiotropy of high degree nodes has on functional predictions” ^9^. We showed previously GeneMANIA’s results (with the normalization) are strongly affected by node degree, and indeed a substantial fraction of performance as measured by ROC curves could be explained by node degree effects ^1^. Likewise, Verbeke *et al.* (2013) describe a gene prioritization method based on local networks that they “assume” reduces the effect of hubs, but provide no direct test ^11^. As we have documented ^1^, various attempts to modify networks to reduce extremes of node degree at best hide the problems from detection. We are open to the possibility that the approach of Verbeke *et al.* has the desired effect.

## Finding better algorithms

In the last year, there have been several interesting evaluations of gene function prediction methods. Our interest lies less in which method does best than in what these evaluations expose about the state of the field as a whole.

Börnigen *et al.* (2012) performed a comparison of eight disease prioritization tools on a set of 42 disease genes ^12^. The task was to prioritize the correct candidate, given whatever input the method requires (typically involving definition of a training set of genes already associated with the target function, and often a list of ~100 candidates in a genomic interval as starting points rather than a genome-wide list). The results were evaluated with ROC curves and with true positive rates at a given threshold. The authors’ method, Endeavour ^13^, was among the top evaluation performers. No evaluation of the impact of multifunctionality was undertaken, but our experience suggests that multifunctional genes tend to be prioritized by these types of methods ^14^. The problem of multifunctionality biasing prioritizations may be at least partly due to the difficulty of obtaining less biased training data. The “known genes” are often going to be biased towards highly-studied genes which do not form a sufficiently specific starting point for making functionally specific predictions. Regardless, Börnigen *et al.* were unable to clearly distinguish a best or poorest method, and the reasons for differences were not identified; it was speculated that differences in the underlying data used were important.

The more ambitious Critical Assessment of Functional Annotation (CAFA) ^15^ was set up in a model very similar to the (now discontinued) function prediction component of CASP6 and CASP7 ^16, 17^. Participating groups predicted GO annotations for poorly-annotated proteins, followed by a waiting period during which some of the targets happened to be annotated by GO curators. The submitted algorithms were then assessed for correctness, relying primarily on a novel gene-centric metric that allowed partial credit for predicting a “similar” term, based on proximity in the GO term graph. Unfortunately, it emerged that this metric led to many methods (including BLAST) being outperformed by a naïve ranking of functions by prevalence (e.g. simply predict functions which are common overall; this approach ranks third or fourth in molecular function prediction), leading the organizers to exclude some results ^15^. Radivojac *et al.* (2013) concluded that simple sequence analysis methods such as BLAST perform poorly, while more sophisticated methods based on integrating diverse data types are a substantial boon. However, in our separate assessment of a substantial portion of the CAFA data, we found that by more conventional metrics BLAST was among the top performers ^18^.

Our concerns about how function prediction works are further supported by a closer inspection of the methods that did well in CAFA. The best performing of them frequently have embedded in them aspects of the naive scoring method; that is, they successfully use knowledge of GO structure and term prevalence. In combination with the gene-centric evaluation metric, this creates a misleading impression of predictive power, in much the same way that a cold-reading mentalist can exploit the prevalence of names and medical conditions to impress a gullible audience. It was also possible to benefit from existing annotations for the targets (hot reading ^18^). While successful in the narrow confines of the assessment, if put to use such approaches would only serve to increase the already strong biases in GO. In other words, in a sense CAFA turned out to be less about predicting gene function than about predicting which proteins would be annotated by GO curators and with which terms.

One of the major issues faced by CAFA is how to operationalize “function”. They (understandably) pass the buck on this issue and take the GO as an appropriate way to define function, with a number of consequent problems. In contrast, DREAM ^19^ is a set of critical assessments motivated, like CAFA, as a means of understanding and improving inference methods (particularly network based ones), but DREAM largely focuses on more specific problems with associated datasets. One DREAM assessment we found interesting (even though it is not strictly about function prediction) focused on breast cancer survival analysis, with the goal of using molecular data (expression and genomic copy number) to improve prediction beyond that provided by clinical features. While a number of performance comparisons were made, one result was that the baseline method - simple Cox regression on clinical features – outperforms most methods across most conditions, even when they include the use of the molecular data. In only 10 out of 28 submissions were models incorporating molecular feature data with clinical able to outperform the baseline clinical predictor. On the one hand, this is encouraging: molecular data may be able to contribute something. On the other hand, the very best method using clinical data only was very close in performance to the best performing method using combined data. As is typically the case in machine learning, ensemble methods performed well (this would also have been true in CAFA), although investigator-based choices also appear to have been critical, since the class of purely automated methods performed particularly badly. In addition, the control of incorporating random gene signatures (or generic/multifunctional ones) with clinical data appears not to have been attempted (as might be suggested by previous research ^20^), with permuted case labelling serving as the negative control instead. This leaves open the possibility that to the extent molecular data is of any predictive value at all, it does not provide us with any guidance as to molecular mechanism (by singling out relevant subsets of genes).

The last assessment we consider is the Critical Assessment of Genome Interpretation (CAGI), which focuses on using sequence data to predict elements of clinical or molecular phenotypes. While the results have not been published formally, the data presented on the CAGI web site are informative ( https://genomeinterpretation.org/). The issue of appropriate controls again rears its head. For example, in the 2011 “personal genome project” assessment of phenotype prediction, the top performing submission appears to have primarily obtained performance by predicting that rare phenotypes would not occur (“due to predicting absence of rare characteristics”). In another competition, ROC curves for predicting Crohn’s disease from exome data appear to show close to half of teams performing below random (although not significantly so, apparently due to low sample size).

Why are critical assessments done? An admirably thoughtful discussion of algorithm comparisons noted that most scientists read new papers thinking “well, of course they say their method is better, but…” ^21^. In part, critical assessments were intended to solve this problem: to help us move forward by making truly representative comparisons. It is not clear this is what is happening for function prediction assessments. Instead, we now have a system where researchers agree to participate and organizers have an obligation not to embarrass them. Thus, often only the top-performing methods are discussed, and the organizers have the same capacity to tweak as the original algorithm developers would have, and many of the same incentives. Discovering that most methods perform quite badly should be headline news, but could reduce enthusiasm for participation to the point of killing off future assessments. This is the usual problem of negative results, but scaled up to apply to the whole field through “consensus”. Perhaps publications of critical assessment should devote equal space to characterizing why methods failed; DREAM’s characterization of the poor performance of molecular data offers a toehold on this issue.

To summarize this section, because there are decreasing returns in tweaking methods, in our view comparisons of algorithms are less important than asking if they work at all and if so, how. Unfortunately this is often very difficult to discern from most of the work that has emerged in the last year, which often vary data as well as algorithms, and do not provide enough information to judge potential drivers of performance such as multifunctionality effects.

## Using prior knowledge

As we noted last year, gene function prediction shouldn’t simply reduce to information retrieval, at least not unwittingly. Organizing existing knowledge and finding overlaps is useful, but is not the principal motivation of network-based methods, which are intended to find novel features in rich data. One way of drawing a distinction is that information retrieval GBA does not as readily suggest novel experiments. Normally, using some experimental feature to draw a functional conclusion suggests that one should try perturbing that experimental feature and observing the result; this will seem redundant if the feature is purely a property of the way the data was explicitly organized. However, the influence of prior knowledge is often hard to discern in the output of prediction methods, so information retrieval can masquerade as *de novo* function prediction.

It is important to realize that methods motivated by information retrieval are still forms of GBA, and are subject to the same potential problems. For example, Hoehndorf *et al.* (2013) created a network of genes based on semantic similarity of phenotypes of genetic diseases and animal models of diseases ( PhenomeNet) ^22^. They then use sets of genetic disease genes from various human databases, and their orthologs in mouse, to evaluate the relevance of this network for identifying gene-disease associations. For example, they rank mouse genes by the similarity of their mutant phenotypes to a target human disease’s phenotypes. They claim their approach is not GBA because “it does not require prior knowledge of the genetic basis of diseases for its predictions”. This is incorrect: because their method uses associations (semantic similarity) and infers “guilt” (involvement of a gene in a disease) based on this, it is obviously GBA, albeit a simple one where the prediction algorithm is a simple ranking of nodes by similarity. The authors may have been hoping that they don’t need to worry about node degree effects and multifunctionality, but we disagree. Using semantic similarity to identify diseases that resemble mouse models seems reasonable; using this to predict disease genes is most definitely GBA and suffers all the same potential pitfalls (and then some).

To see why, we note that the nodes in the network used by Hoehndorf *et al.* can be regarded as the set of both genes and diseases/phenotypes with edges indicating high semantic similarity across phenotypes. A disease node then provides the training data (a set of associated genes), and nearby gene nodes are the predicted relevant genes. By taking the gene-centred data (mutants, etc.) and treating it as equivalent to disease the authors are incorporating a hypothesis in addition to GBA, not instead of it. That is, a disease is treated conceptually as if it was gene-like. Consider that the same model should work if we were trying to predict effects of mutations from other known mutation effects through cross-validation, which would then be GBA (but also including an information retrieval component).

The use of various flavors of annotation similarity to build or influence networks is already endemic in function prediction, as we noted previously. A recent example is the work of Youngs *et al.* (2013), who use information on GO annotations to compute priors for function prediction ^23^. In this manner, the likelihood that a gene is predicted to be annotated with a certain GO term is influenced by whether its other annotated GO terms tend to co-occur with desired GO term. This method, which is influenced by early work ^24^ performs strongly in cross-validation, but we see two issues. The first is that, once again, the authors claim their evaluation approach addresses biases we have reported, without providing evidence. Second, it treats annotation biases as something to exploit (somewhat like Singh-Blom *et al.*), which we regard as a shaky proposition when it comes to predicting gene function, as opposed to performing information retrieval.

One interesting feature of the attempts to improve networks, methods, and priors in GBA is that researchers in each area can take the other area to be a gold standard. Thus, researchers focusing on protein interaction networks may use GO to obtain “better” interaction data ^25^. Conversely, researchers wish to treat the network as a gold standard to improve GO ^26, 27^. In the meantime, algorithm developers treat both networks and annotations as a gold standard when comparing methods. In all these cases, researchers are performing what we would call “GBA” and working with the alignment between how genes form groups (using some method) as characterized in data and how those genes are grouped by prior annotations. The fact that there is some form of alignment is repeatedly rediscovered. A problem we perceive is that the duality of the gold-standards is increasingly blurring the lines between predictions and data. For example, Dutkowski *et al.* (2013), when benchmarking their method against GO, initially used as input some networks that were influenced by data from GO (e.g., YeastNet ^25^), and so had to perform separate experiments to remove this confound. Similarly, the work of Magger *et al.* discussed above used data on disease gene expression patterns ^28^ that were derived in part from the same protein interaction data that Magger *et al.* then use to perform tissue-specific predictions, though the implications of this are unclear. Recently we documented how protein interactions and gene ontology annotations are in many cases derived from the same publications ^29^. Data resources used in genomics are becoming more intertwined, so ever greater care is required to avoid contaminating computational experiments with unwanted biases.

## GBA success stories?

Guilt by association is widely agreed to be a valid method for investigating gene function. As mentioned, our concerns largely have to do with how GBA is performed and evaluated computationally (though the biases in existing knowledge could have impact on GBA even when it is conducted by hand). We also want to know, when GBA does work, is it because of “generic features” such as node degree, or are the GBA methods working the way most computational biologists hope they are working, which is inferring specific things about a gene based on specific features of its network neighbors. It is therefore of great interest to us and the rest of the computational GBA field to see use of GBA “in the wild”.

Our review of the literature reveals different stories for disease gene prioritization and for other function prediction tasks. It is uncommon to see papers that report using computational GBA as an important means of identifying genes with a desired function (ignoring the role of sequence similarity, which is no doubt the most-used GBA method; methods that use more complex network-based approaches are our focus here). In contrast, genetics researchers faced with a genomic interval or a set of candidates seem to more readily turn to prioritization tools for assistance. This may be because the task of prioritizing a few genes (often 10–20) is simpler than prioritizing the entire genome, or that the task of identifying a disease gene is more clearly defined.

To take a well-known method as example of how algorithms are used in practice, GeneMANIA is implemented in a web-based tool described by its developers as a gene recommender system – essentially, using GBA ^9^. A survey of recent citations suggests that the function prediction aspect is not the focus of most users of GeneMANIA ^30– 34^; in some cases users express an interest in predicting interactions, but not functions ^35^. This may be because GeneMANIA’s tools do not operate on functions as usually defined in GBA settings; they take as an input a set of genes chosen by the user, and show a gene network with nodes selected using the GeneMANIA algorithm. In this way it is very similar to STRING ^36^ and HumanNet ^37^, which generate gene networks, which are adjusted by their agreement with inputs such as GO. We suspect some users of GeneMANIA, STRING and HumanNet do not realize that they are looking at the output of a guilt-by-association-influenced approach.

This is not to say that computational methods are not directly used successfully for function prediction. But even in such cases, it is often very difficult to determine how exactly GBA worked. Users of GBA are justifiably relatively uninterested in how they get to an answer, only that it is correct and leads to a new set of testable hypotheses or insights. Thus GBA success stories tend to be somewhat light on details and heavy on ad hoc aspects. We briefly mentioned several success stories in our original commentary. Some additional examples have since come to light and bear discussion.

Tacutu *et al.* (2012) provide a valuable rare large-scale assessment of a computational GBA task ^38^. They wished to predict genes involved in regulating the longevity of *Caenorhabditis elegans*. Their input is a set of 205 known longevity-associated genes (LAGs) in a worm protein interaction network of 871 genes. A similar network was constructed for human orthologs. They then made predictions in a simple way, considering any gene that was a network neighbor of known LAGs (or orthologs), limited to the subset of candidates that are required for development (essential genes). This yielded 500 candidates of which 374 were tested. They report 19 of these validated, a success rate of 5%, compared to a rate of 2.4% based only on genes critical for development. It is notable that most of the predictive information came from exploiting the prior knowledge that essential genes were good candidates: the success rate went up five-fold from ~0.5% (for genome-wide screens) to 2.4% for essential genes, whereas adding the protein interaction data increased success by another two-fold. Tacutu *et al.* do not report any information on potential node degree effects, but obviously the largest number of candidates would have to come from the highest node-degree LAGs given the simplicity of the method. We suspect that many GBA experts would want to know how it would have worked with “better data” and a “more sophisticated algorithm”.

Putnam *et al.* (2012) sought to identify yeast genes suppressing genomic instability ^39^. Their initial input was 75 genes already known to be involved in genomic instability, and 928 genes in which mutations cause sensitivity to DNA damage. This set was expanded by choosing genes which have similar profiles of genetic interactions – this appears to be their key GBA step. The final set of genes numbered 1041, which were further prioritized in a second GBA-like step, based on their genetic interaction profiles with known DNA damage response genes, and by apparently manual selection, leading to selection of 87 genes for experimental follow-up. Of these, 40% had a detectable effect on genomic stability when mutated. This is impressive, but it is difficult to quantify the contribution of computation versus manual selection, and relatively few candidates were entirely novel. The authors speculate that their success rates might have been even higher if they had genetic interaction from a more relevant phenotype. For example, many genes that clustered with known DNA damage-related genes – and which thus looked “guilty” - failed to validate. As in the case with Tacutu *et al.*, we note the relatively simple data used and the simple approach, combined with hand-tuning.

We have also reviewed some recent applications of disease gene prioritization tools, which as we comment above are seemingly used more commonly than function prediction tools (even though they are conceptually similar). We are struck by two trends. First, many (perhaps most) papers that apply such methods make no strong conclusion as to whether they have found the right gene ^40– 48^. That is, the results are treated a bit like GO enrichment analyses: as suggestive or exploratory. Second, some papers that report prioritization tool results supplant them with more precise or manually-identified information, such as the existence of an orthologous mouse mutant that has similar phenotypic features ^49– 52^.

We note a few trends from these reports. Black-box application of existing prioritization methods played at best a supporting role. The use of custom methods for creating initial target sets were important, sometimes based on experiments under the investigator’s control, rather than existing annotations from public databases. Data that was specific to the biology was deemed important: using generic data is a fallback. Not surprisingly, even with these ingredients, success in converting computationally-prioritized genes into documented hits is far from guaranteed. And while these examples bolster the claim that GBA can work, exactly how they are working with regards to multifunctionality bias is still left unclear.

## Conclusions

A theme that emerges from our review is brought out by the difference between the practices of computational biologists and those who actually use function prediction tools (loosely defined). These differences should come as no real surprise, but it has important implications that we feel are not being attended to sufficiently. It may be that biologists are happy with high-quality information retrieval tools, and are not actually very interested in function prediction at all. That creates a difficulty for those who are interested in predicting function, who feel compelled to develop new methods to do so, and who want their tools to be used by others. Such practitioners are left to test their methods on the GO, which we are increasingly certain is a waste of time, in the sense that it isn’t realistic, it isn’t what interests biologists, and it is easily confounded with the data used for prediction. It remains difficult to tell when methods are actually doing something useful, because evaluations have been weak, and the “in the wild” uses are obfuscated by various hand-tunings, publication bias, or inadvertent cherry-picking.

We regard these as major issues, but this is a far cry from disagreeing with functional inference overall. Some authors appear to have interpreted our papers as concluding that GBA is useless ^53, 54^, but this is too broad a brush. We fully believe in the GBA principle, and computational methods can be useful. The difficulty is in telling *when* and *how* they are working. The concern about “when” is summarized by our findings that cross-validation analysis can be very misleading – to the point of being potentially irrelevant - for predicting future performance ^2^. The concern about “how” is reflected in our demonstrations that gene multifunctionality and node degree effects are often more important in determining the outcome of a GBA analysis than details about the connections in the network ^1^. These two realizations should affect practice, but they do not mean that the predictions one makes are always incorrect.
