# Supplementary material for: Progress and challenges in the computational prediction of gene function using networks: 2012-2013 update
Source: F1000Res. 2013 Oct 31;2:230. [Version 1] doi: 10.12688/f1000research.2-230.v1 (PMC3962002; doi:10.12688/f1000research.2-230.v1)
Supplement: Peer review history

# Peer review: Referee response for version 1

**Published:** 2014-02-05

**Authors:** Vivek Anantharaman

**Affiliations:** 1 National Center for Biotechnology Information, National Library of Medicine, National Institutes of Health, Bethesda, MD, USA

The authors have discussed the pitfalls of automated GBA and ways to improve functional prediction. Automated methods for GBA are only as good as ontologies and curated reference datasets. Ontologies like GO suffer from poor quality annotation being propagated throughout their data that result in “Garbage in – Garbage out” phenomenon. A generic functional prediction is the best one can expect from existing automated methods.

From my experience, I have found that accurate functional prediction requires a mix of local sequence similarity and sequence profile searches, proper sequence analysis with study of sequence and phyletic conservation, structural analysis, network studies of various data points, and correlation with experimental data, all done with a heavy dose of manual tuning. None of the automated methods of GBA give consistent accurate prediction, without manual intervention.

The review laid out by the authors is a good analysis of the challenges and limitation of gene function prediction. One area that the authors do not explicitly discuss is the great difference between eukaryotes and prokaryotes. GBA is currently far more effective in the latter due to operons, which are not available in eukaryotes.

I have read this submission. I believe that I have an appropriate level of expertise to confirm that it is of an acceptable scientific standard.

# Peer review: Referee response for version 1

**Published:** 2013-11-08

**Authors:** Stefano Toppo

**Affiliations:** 1 Department of Molecular Medicine, Università degli studi di Padova, Padova, Italy

This opinion article deals with the long standing issue of protein function prediction in its broader sense. The authors express an interesting and most of the time shareable point of view about the negative impact of gene multifunctionality that influences gene network-based guilt-by-association studies.

The paper is really well written and organized in sections that focus on different aspects of function prediction and its pitfalls. Nonetheless, there are some minor points that I would suggest mitigating, as they sound too harsh and are, as far as I’m concerned, partly incorrect.

In the “ *Finding better algorithms*” section, CAFA is mentioned and commented on but I would like to pinpoint some aspects about how this is done and some related issues (below). Even the glorious series of CASP experiments, that the authors have mentioned, suffered a lot in their first editions but what is more important is that both assessors and participants are aware of this and that improvements are planned, as far as I know.

The prediction results from the CAFA experiment could perhaps be framed around some different points of view:

**1:**

Looking at the F-measure results for top performing methods there is little to be happy about. There are the following additional issues coming out from CAFA: are we really sure that some of the predicted functions are not correct? Is this rather an effect caused by the possible incompleteness of some experimental data? In other words, can anyone assert firmly that there is nothing else to discover about the function of a protein? I would definitively say NO. There is more than meets the eye and besides, the benchmark is incomplete by definition as it will never complete in the future either, no matter what information is added. Not only that, but even novel experimental evidence can turn false positive predictions of protein function into a true positive. On the flip side, true positive predictions can equally be refuted by fresh experimental data and consequently turn into a false positive.

 

**2:**

Some functions of the CAFA experiment were extremely difficult to predict and hard to “guess” in any way, both from a simple sequence similarity approach or other more sophisticated techniques based on machine learning. In summary, some CAFA targets were not so easy to predict.

 

**3:**

Additionally with CAFA, much marginally informative experimental data was collected for many targets that mainly derived from PPI experiments. The term under indictment is “protein binding” which is heavily present, for example, in the GOA database of annotated proteins. The assessors’ decision to discard “protein binding” in the final evaluation was, consequently, correct. The good performance of naïve and BLAST methods (when “protein binding” is considered in the assessment) depends on the high occurrence (multifunctional?) of one function in the database and its prevalence over the others so that it is very easy “to predict”. In this sense, it is not exactly correct to assert that BLAST and naïve are (almost) the best performing tools because many tools participating to CAFA, whenever possible, tried to provide more informative annotations in place of the less informative “protein binding”. Taking this into account, “protein binding” would have been inappropriate to use in the final evaluation because it would have rewarded BLAST and naïve methods artificially but penalized others.

In contrast, the main issue is that databases contain biased annotations and a few scarcely informative terms dominate the scene. In this respect, I totally agree with the authors that multifunctionality poses serious problems to function prediction algorithms. So how might one mitigate the effect of multifunctional and scarcely informative annotations? 

Perhaps CAFA will need to settle in the next editions and the contribution to this process of renewal should be constructive and proactive rather than purely critical.

 

**4:**

The authors recognize that successful stories may be limited, simple and hand-tuned. Reverse engineering results is demanding and I agree with the authors, but I would note that the excess in the analysis, as suggested in the when/how methods perform section, could lead to an overestimate/underestimate of the behavior of the tools and miss their general action. As a matter of fact, biology is made of more exceptions than rules and tools are designed to follow only the rules. Can the authors suggest some possible ways in which a tentative solution can be set up that could be discussed and adopted in critical assessments of function prediction tools?

 

 

**5:**

To me, the distinction in this paper between between GO and protein annotations using GO are not clear enough. I would, for instance, rephrase the following: 

“…. *We also suggested that part of the problem is the reliance by computational biologists on gold standard annotations such as the Gene Ontology ….”*

To something like:

“…. We also suggested that part of the problem is the reliance by computational biologists on gold standard annotations such as the Gene Ontology Annotation database (GOA) ….”

I know the authors know the difference between the two and for this reason I would recommend that they clarify this aspect and do not confound what GO and its countless instances are (one of them is GOA).

As an obvious reminder, GO is an abstraction of the knowledge tentatively organized in a directed acyclic graph and is a controlled vocabulary intended as the rosetta stone of different interpretations and expressions of the same concepts. I strongly believe that GO is rigorous and we can trust it. On the contrary, GOA contains GO instances used to describe proteins. Using the metaphor of programming language, the GO term is the “object” and its use in GOA, or other databases containing GO annotated proteins, is the “instance” of that “object”. This is an important difference because the “object” is abstract and may be varied in a number of ways that can be right or wrong when thinking about protein annotation. It is not the GO term definition per se in the dock but rather its utilization as a descriptor of protein function stored in public databases. The authors already published a paper on GO based annotations and their distribution in GOA over time. In other words, one can rely on the GO descriptions and their positions in the graph (though GO is continuously revisited, it is rather stable) but must pay attention to the proteins annotated with GO terms because they can be inappropriate and change over time, as already evaluated by the authors in a previous work (indeed, GO annotations in GOA change frequently).

It would be very interesting to know what the authors think about the latter phenomenon, i.e. the updating of old annotations and their syncing with novel and more precise GO ontologies.

Most of the issues, fully and carefully described by the authors, may be due more to this aspect than others. Generic annotations may have been used at the beginning of the story when GO was still incomplete and with poor coverage of biological knowledge. Thinking about “Inferred from Electronic Annotations” (IEA), these GO terms may have created the multifunctional phenomenon as they had time to spread and have consequently become both pervasive and difficult to eradicate or update.

I have read this submission. I believe that I have an appropriate level of expertise to confirm that it is of an acceptable scientific standard.
